# Muscle Activation Patterns During Movement Attempts in Children With Acquired Spinal Cord Injury: Neurophysiological Assessment of Residual Motor Function Below the Level of Lesion

**DOI:** 10.3389/fneur.2019.01295

**Published:** 2019-12-20

**Authors:** Darryn A. Atkinson, Laura Mendez, Natalie Goodrich, Sevda C. Aslan, Beatrice Ugiliweneza, Andrea L. Behrman

**Affiliations:** ^1^Doctor of Physical Therapy Program, University of St. Augustine for Health Sciences, Austin, TX, United States; ^2^Kosair Charities Center for Pediatric NeuroRecovery, University of Louisville, Louisville, KY, United States; ^3^Kentucky Spinal Cord Injury Research Center, University of Louisville, Louisville, KY, United States; ^4^Pediatric Neurorecovery Program, Frazier Rehab Institute, Louisville, KY, United States; ^5^Department of Neurological Surgery, University of Louisville, Louisville, KY, United States

**Keywords:** pediatric spinal cord injury, neurophysiology, electromyography, rehabilitation, motor assessment

## Abstract

**Introduction:** Characterization of residual neuromotor capacity after spinal cord injury (SCI) is challenging. The current gold standard for measurement of sensorimotor function after SCI, the International Society for Neurological Classification of Spinal Cord Injury (ISNCSCI) exam, seeks to determine isolated intentional muscle activation, however many individuals with SCI exhibit intentional movements and muscle activation patterns which are not confined to specific joint or muscle. Further, isolated muscle activation is a feature of the neuromuscular system that emerges during development, and thus may not be an appropriate measurement standard for children younger than 6.

**Methods:** We utilized neurophysiological assessment methodology, long studied in adult SCI populations, to evaluate residual neuromotor capacity in 24 children with SCI, as well as 19 typically developing (TD) children. Surface electromyography (EMG) signals were recorded from 11 muscles bilaterally, representing spinal motor output from all regions (i.e., cervical, thoracic, and lumbosacral), during standardized movement attempts. EMG records were subjectively analyzed based on spatiotemporal muscle activation characteristics, while the voluntary response index (VRI) was utilized for objective analysis of unilateral leg movement tasks.

**Results:** Evidence of intentional leg muscle activation below the level of lesion was found in 11/24 children with SCI, and was classified based on activation pattern. Trace activation, bilateral (generalized) activation, and unilateral or isolated activation occurred in 32, 49, and 8% of movement tasks, respectively. Similarly, VRI analyses objectively identified significant differences between TD and SCI children in both magnitude (*p* < 0.01) and similarity index (*p* < 0.05) for all unilateral leg movement tasks. Activation of the erector spinae muscles, recorded at the T10–T12 vertebral level, was observed in all children with SCI, regardless of injury level or severity.

**Conclusions:** Residual descending influence on spinal motor circuits may be present after SCI in children. Assessment of multi-muscle activation patterns during intentional movement attempts can provide objective evidence of the presence and extent of such residual muscle activation, and may provide an indicator of motor recovery potential following injury. The presence of residual intentional muscle activation has important implications for rehabilitation following pediatric-onset SCI.

## Introduction

Neuromotor control of movement is a complex process, requiring multiple descending, and ascending systems to integrate sensory input and coordinate motor output sufficient for the simultaneous maintenance of posture and balance in both static and dynamic (i.e., gait) contexts (1–3). As in adults, spinal cord injury (SCI) in children causes a loss of motor and sensory function below the lesion due to the disruption of these ascending and descending projections between supraspinal and spinal networks.

Accordingly, assessment of residual motor and sensory function below the level of lesion is a critical determinant of the recovery prognosis and subsequent access to rehabilitative therapies (4). The current gold standard for measurement of residual sensorimotor function after adult SCI is the International Society for Neurologic Classification of Spinal Cord Injury (ISNCSCI) exam, also known as the American Spinal Injury Association (ASIA) Impairment Scale (AIS) (5). The ISNCSCI motor exam evaluates intentional, isolated muscle activity in 5 key muscles of the arms and legs, the results of which have proven useful in the determination of recovery prognosis following SCI in adults (4, 6–9). However, isolated movement represents only one aspect of neuromotor control; the ISNCSCI exam was not designed to assess residual motor control resulting in movement or muscle activation at multiple joints, which may be present after neurological injury. In addition, the ISNCSCI was not designed for use in children, and is not valid in children younger than 6 years of age (10, 11).

Characterization of residual neuromotor control after pediatric-onset SCI is challenging; children of different ages represent different levels of neuromuscular maturation, adding an additional layer of complexity. The development of neuromotor control in children is a non-linear process which can vary substantially in terms timing, rate, and extent across tasks and among children of the same age. Different aspects of development (i.e., gross vs. fine motor control) emerge at disparate chronological ages and occur at different rates. Specifically, children aged 6 and younger demonstrate substantial interindividual variability in the performance of motor tasks (12, 13). Young children may exhibit widespread muscle activation during performance of a given task, and frequently lack the ability to produce the isolated or coordinated muscle activity (14–18). Therefore, isolated muscle activation may not be an appropriate measurement standard for children younger than 6. There are no existing standardized assessments available which can reliably assess motor and sensory function after SCI in all children. As a result, little is known regarding the extent of sensorimotor function which may persist following SCI in children, especially those under the age of 6.

To date, evaluation of neuromotor control in children has largely been limited to observational scales (19, 20). While these methods are sufficient for the gross evaluation of movement performance and may be easily used in clinical settings, distinctions between intentional movements, unintentional or “reflexive” movements, and “spasms” can be difficult (19). As recovery-focused interventions continue to be developed and improved (21–26), determination of the presence of residual supraspinal-spinal connectivity after SCI has clear implications for rehabilitation goals and outcomes. There is an important need for age-based assessment of sensorimotor function in children with SCI.

The goal of the current study was to assess the capacity for residual muscle activation below the lesion in the developing and injured nervous system utilizing neurophysiological assessment methodology. We hypothesized that children with SCI retain the ability to produce muscle activation below the level of lesion during intentional movement attempts which can (i) be characterized and understood in the context of motor control development and (ii) provide evidence of residual supraspinal-spinal connectivity after SCI.

In order to generate an objective profile of residual motor capacity, we developed a standardized neurophysiological assessment to evaluate the pattern and extent of spinal motor activity generated during the performance of standardized movement tasks in children with neurological injury/disease. The current methodology has long been utilized in adult SCI research (27–32). This methodology was developed specifically to discriminate between muscle activity which can be repeatedly observed in response to a specific movement attempt under standardized conditions, and those which are the result of fasciculations or myoclonic activities. The strengths of this assessment are its objectivity, standardization, and ability to assess multi-segmental spinal motor output during specific movement attempts, thus allowing identification of multi-segmental patterns of motor output beyond the presence or absence of isolated activity in a single muscle (33, 34).

## Methods

### Participants

A sample of convenience was recruited from the Pediatric Neurorecovery clinic at Frazier Rehabilitation Institute as well as from the local community and enrolled in this study. All participants aged 7 and older gave written informed assent, and consent was obtained from their parent or legal guardian for all participants under 18 (University of Louisville IRB #15.0183). Children between the age of 1–21 years were invited to participate. Participants with non-progressive SCI in stable medical condition were enrolled, while those with progressive etiology, ventilator dependence, unhealed fracture, pressure sore, or current urinary tract infection were excluded. Injury severity was assessed via the International Standards for Neurological Classification of SCI (ISNCSCI) American Spinal of Injury Association (ASIA) Impairment Scale (AIS) (5) in children 6 and older (10, 11). None of the children included in the current study presented with clinical signs of unilateral spinal injury such as Brown-Sequard syndrome (35), in which unilateral motor and contralateral sensory function is observed. For all patients, level of spinal injury was determined based on review of medical records including vertebral fracture levels in the case of traumatic injury and MRI reports identifying areas of damage in non-traumatic subjects. Children currently taking anti-spasticity medications such as baclofen and children who had received botox injections within the last 3 months were excluded from this study.

### Electromyographic (EMG) Recording and Data Collection

EMG was recorded with wireless, pre-amplified electrodes (Cometa®) with a 2,000 Hz sampling rate and a bandpass filer of 10–500 Hz. After preparation of the skin with alcohol, EMG electrodes were placed mid-belly (36) using self-adhesive 1.25” disposable electrodes. Muscles, all assessed bilaterally, included upper trapezius (UT), anterior deltoid (DELT), rectus abdominus (RA), erector spinae (ES), adductor (ADD), gluteus maximus (GLUT), rectus femoris (RF), vastus lateralis (VL), medial hamstrings (MH), tibialis anterior (TA), and medial gastrocnemius (MG). EMG recordings were annotated according to the protocol. Activity outside of the designated protocol, the Functional Neurophysiological Assessment (FNPA), such as: coughing, spasms, and extraneous movement, was noted and excluded from further analysis.

### Functional Neurophysiological Assessment (FNPA) Protocol

**Figure 1:**
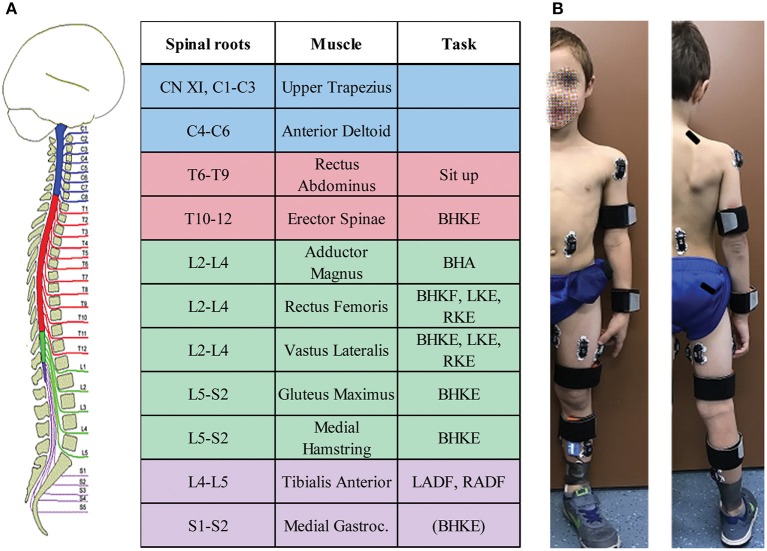
Pediatric functional neurophysiologic assessment summary. **(A)** Muscles from which EMG is recorded during the assessment, and their respective spinal root innervation. The right-hand column lists the movements used to assess intentional activation for the applicable muscles. **(B)** Example of electrode placements. Rectus Abdominus electrodes were placed para-umbilically, while erector spinae electrodes were placed with the superior aspect of the electrode at the level of the T10 spinous process. Written informed consent was obtained from the child's parent for the use of this image.

The standardized protocol was modified from the FNPA assessment (32) with fewer movement tasks included to reduce the overall length of the assessment. With children as participants, it was necessary to reduce the experiment length to within 2 h for successful participation and completion. The number of muscles from which EMG signals were recorded was also reduced due to the smaller size of children's extremities (Figure 1). Recorded activities started with 2 min relaxation in supine, followed by intentional movement trials. For each movement, a minimum of three attempts were performed on the examiner's verbal cue, each lasting a minimum of 3 s. In preparation for each maneuver, the examiner first described and demonstrated the movement. For each trial, the examiner provided verbal cues to start, continue, and stop the movement attempt. Instructions and verbal cues were adapted as needed between participants, based on patient age and engagement level. Intentional movements assessed included: unilateral knee extension (left and right, LKE and RKE), unilateral ankle dorsiflexion (left and right, LADF and RADF), bilateral hip and knee flexion “knees to chest” (BHKF), bilateral hip and knee extension (legs on bolster) (BHKE), bilateral hip adduction (BHA), neck flexion (NF), and sit up. Video and audio of each assessment were recorded.

### Data Reduction and Analysis

#### Qualitative Assessment

All experiment videos were viewed and checked against notes taken at the time of assessment for absence/presence of EMG activity and movement. Motor recruitment patterns, as assessed by the number of muscles generating EMG activity as well as the amplitude of activity, were reviewed and categorized by a physical therapist evaluator and coded as: N = no activation, T = trace activation, B = bilateral (generalized) activation, U = unilateral activation, and I = isolated activation. EMG activity patterns for BHKF, BHKE, BHA, NF, and sit up were coded using scores N, T, and B, based on the bilateral nature of the movement. An increase in EMG amplitude in any muscle, observed by visual inspection and occurring during each trial for a given movement, was considered as intentional muscle activation. During evaluation of EMG results, any task in which EMG activity occurred after some time delay from the onset of the movement attempt was noted. Delayed muscle activation was operationally defined as a difference in time of at least 1 s between the onset of EMG activity in muscles deriving their innervation from spinal segments above the level of injury (onset of movement attempt), and the onset of EMG activity in muscles innervated below the level of lesion (cf. **Figure 5**). Results were compiled for each patient across trials (see **Table 3**).

To avoid misidentification of myoclonic activities or fasciculations as intentional muscle activation during the movement tasks, several strategies were utilized (31, 37). First, background EMG with the child at rest was collected in each participant prior to attempting movement tasks. This allowed for observation of the presence of any form of spontaneous muscle activity, including its frequency and muscles in which it may be present. Study participants were instructed to completely relax prior to the onset of each movement attempt, and relaxation in the studied muscles was confirmed and monitored in real time through ongoing visualization of all EMG channels during acquisition. If increased EMG activity was noted as compared with the background resting EMG acquiring at the start of the study, the examiner waited until this activity ceased prior to continuing. Performance of each task was initiated through a verbal cue from the examiner, once relaxation in the studied muscles was visualized. Each task was performed three times, and EMG activity was compared across all three trials. Muscle activity was considered as intentional only when it occurred following the command to begin, and was observed in each trial for a given movement task.

#### Quantitative Assessment

The time interval between the onset of each movement attempt (as defined in the previous section) and the examiner's cue to end the movement was used to identify the EMG signals to be analyzed from each task and attempt. When no EMG was present, the start and end of the attempt were selected according to the examiner cue, and the observable effort and relaxation of the participants.

The Root Mean Square (RMS) of the EMG activity was calculated for each muscle and time interval, then averaged across the three trials for each movement task. Further examination of multi-muscle activation patterns was then undertaken using the voluntary response index (VRI) methodology (33, 34). First, RMS values for each muscle were used to generate a Response Vector (RV) for each movement task, for a total of nine vectors for each participant. Different muscles were used to create the RV for a given movement task, depending on relevance to the movement (33). For bilateral events and unilateral knee extension RA, ES, ADD, VL, RF, MH were considered, creating a 6-element RV. For these events, TA and MG were excluded, since they are not prime movers in the events, and no instruction was provided regarding ankle movement. For unilateral ankle dorsiflexion, sit up, and neck flexion, TA and MG were also included, resulting in an 8-element RV. Leg muscles were included for sit up and neck flexion since trunk flexion movements against gravity are still in development in the pediatric population, and compensatory/stabilization activity of the legs is expected for these movements (38, 39). Next, Prototype Response Vectors (PRVs) were generated for each movement task by averaging the TD group RVs for each movement assessed. All individual RVs from each group were then compared to the PRV. The VRI analysis was performed for nine movements, producing two independent elements: the magnitude and the similarity index (SI). The magnitude of the vector is a representation of the recruitment of motor units (how much total muscle activity was recorded) during the event. The SI represents the similarity in the distribution of multi-muscle activity of a given subject compared with the PRV, and returns values between 0 and 1, where a value of 1 means that the RV had an equal distribution of the activity pattern to the PRV.

#### Statistical Methods

Children's age at the time of the experiment, age at the time of injury and years since injury were summarized using mean with associated standard deviation (SD), median with associated interquartile range (IQR) and full range (minimum to maximum). The Wilcoxon Rank Sum test was used for 2 by 2 comparisons and the Krustal-Wallis test for 3 or more group comparisons. Gender and mechanism of injury were summarized with frequency count and percentage and they were compared with the Chi-square test. Task outcomes measurements (similarity index, magnitude and subjective score) were summarized using means with standard deviation and median with interquartile range. The comparison of these tasks' central location across different groups was performed using Wilcoxon Rank Sum Test. To compare their variability, the Brown and Forsythe's test was used. The correlation between the measurements of the task outcomes was evaluated using the Spearman correlation. The Chi-Square test was used to test the bivariate association of mechanism of injury and presence of intentional muscle activity. Then, the logistic regression was used to evaluate this association adjusted for age. To test whether correlations found were significantly different from 0, the *t*-test with (n-2) degrees of freedom was used on computed values v $=\;\sqrt{\frac{\left(n-2\right)r^{2}}{\left(1-r^{2}\right)}}$, with r being the Spearman correlation and n the total sample size. All tests were 2-sided with a significance level of 0.05. Data analysis was performed in SAS 9.4 (SAS Institute Inc., Cary, NC).

## Results

Nineteen TD children participated, including 9 males and 10 females (average age = 6.9 ± 2.8 years old, range: 3–13); 24 children with SCI participated, including 12 males and 12 females (average age = 7.6 ± 3.4 years old, range 3–15). Eleven children presented with cervical SCI (mean age = 7.8 ± 4.1 years old), as well as 13 with thoracic SCI (mean age = 6.4 ± 2.6 years). Mean age at injury was 3.4 ± 1.8 years, and mean time since injury at the time of assessment was 4.6 ± 3.6 years. No significant differences in age or gender were found between TD, cervical SCI, and thoracic SCI groups (Table 1). Clinical characteristics for each SCI participant are given in Table 2. No significant differences were noted for age at injury, time since injury, or mechanism of injury between cervical and thoracic SCI participants.

**Table 1:** Participant demographics.

	**Descriptive summary**			***p*-value**
	**SCI-cervical**	**SCI-thoracic**	**TD**	
	***n =* 13**	***n =* 11**	***n* = 24**	
**Age (years)**				0.5347
Mean (SD)	7.8 (4.1)	6.4 (2.6)	6.9 (2.8)	
Median (IQR)	6 (5,10)	6 (4,7)	7 (4.5, 9)	
Range, min–max	3–15	4–12	3–13	
**Gender**				0.9334
Male, *n* (%)	7 (53.85%)	5 (45.45%)	12 (50%)	
Female, *n* (%)	6 (46.15%)	6 (54.55%)	12 (50%)	
**Year since injury**				0.2959
Mean (SD)	5 (3.8)	3.1 (3.1)	N/A	
Median (IQR)	3.4 (2.1, 7.9)	1.5 (1, 4.2)		
Range, min-max	0.5-10.9	1-10.7		
**Age at injury (years)**				0.3388
Mean (SD)	3.2 (2)	3.7 (1.6)	N/A	
Median (IQR)	3.3 (2.1, 4)	4.1 (3.1, 4.8)		
Range, min–max	0.2–6.8	0–5.8		
**Mechanism of injury**				0.459
Traumatic, *n* (%)	9 (69.23%)	6 (54.55%)	N/A	
Non-traumatic, *n* (%)	4 (30.77%)	5 (45.45%)		

**Table 2:** SCI participant injury characteristics.

	**Participant**		**Age (yrs.)**	**AIS**	**Time since injury (yrs.)**	**LOI**	**Age at injury (yrs.)**	**Mechanism of injury**
Cervical	P23	M	3	Not valid [Chafetz et al. (10)]	1.0	C2	2.4	Transverse myelitis
	P22	M	4		0.5	T4	4.0	MVA
	P8	F	4		3.4	C2	1.3	MVA
	P20	M	5		0.8	C1-C2	4.7	MVA
	P4	F	5		5.1	C3	0.2	Fall
	P7	F	6	C4 C	2.6	C4	3.5	Ischemic insult
	P14	M	6	C7 C	3.2	C7	3.5	MVA
	P29	F	8	C2 A	2.1	C2	6.3	Medical/Surgical complication
	P10	M	9	C5 C	8.9	C5	0.4	Transverse myelitis
	P5	F	10	C5 C	7.9	C5	2.7	Transverse myelitis
	P30	M	13	C5 A	10.9	C6-7	2.1	MVA
	A98	F	14	C8 A	7.7	C8	6.8	MVA
	P17	M	15	C6 C	10.9	C6	3.3	GSW
	*n = 13*	*M = 7,* *F = 6*	*avg. = 8.2 ± 4.0*		*avg. = 5 ± 3.8*		*avg. = 3.1 ± 2.0*	*T = 9,* *NT = 4*
Thoracic	P3	M	4	Not valid [Chafetz et al. (10)]	4.2	T4	0.0	Neuroblastoma
	P15	F	4		1.1	T12	3.1	Epidural abscess T8-T12
	P32	F	4		1.4	T2-T3	3.3	MVA
	P25	F	5		1.0	T9	4.2	Spinal cord Infarct
	P16	M	5		1.5	T10	4.0	Medical/Surgical complication
	P9	F	6	T2 B	1.0	T4	5.0	MVA
	P13	M	6	T3	1.0	T3	5.8	MVA
	P6	M	7	T7 C	3.5	T7	4.4	MVA
	P18	F	7	T2 A	2.4	T2	4.8	MVA
	P1	M	10	T1 C	6.7	N/A	4.1	Spinal epidural hematoma
	P31	F	12	T3 C	10.7	T12	1.9	Transverse myelitis
	*n = 11*	*M = 5,* *F = 6*	*avg. = 6.7 ± 2.6*		*avg. = 3.1 ± 3.1*		*avg. = 3.7 ± 1.6*	*T = 6,* *NT = 5*
Total	*n* = 24	M = 12, F = 12	avg. = 7.5 ± 3.4		avg. = 4.6 ± 3.6		avg. = 3.4± 1.8	T = 15, NT = 9

*AIS, American spinal injury association Impairment Scale; LOI, level of injury*.

### Muscle Activation Patterns in Typically Developing Children

**Figure 2:**
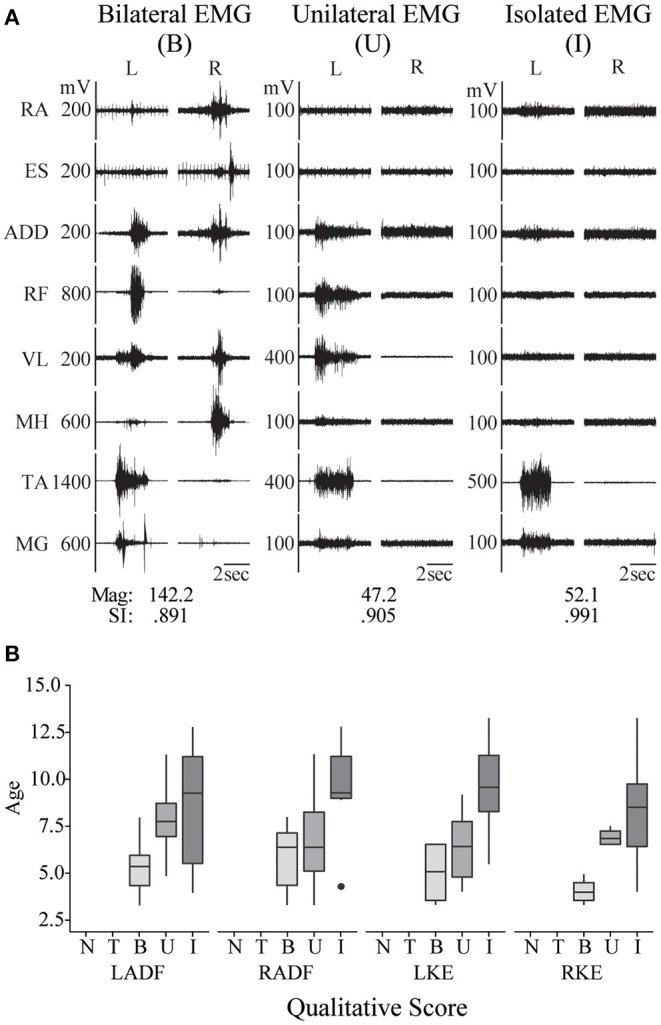
Pediatric functional neurophysiologic assessment: evaluation of typically developing motor patterns. **(A)** EMG recordings from 3 different TD children (aged 3, 7, and 11 years old, left to right) during performance of the left ankle dorsiflexion task, demonstrating the three muscle activation patterns observed during development of isolated muscle activation in these children: bilateral muscle activation, unilateral muscle activation, and isolated muscle activation. RA, rectus abdominus; ES, erector spinae; ADD, adductor magnus; RF, rectus femoris; VL, vastus lateralis; MH, medial hamstrings (semitendinosus); TA, tibialis anterior; MG, medial gastrocnemius. SI and magnitude values corresponding to each example. **(B)** Relationship between age and muscle activation pattern, where N, no activation; T, trace activation; B, bilateral activation; U, unilateral activation; and I, isolated activation. RADF, right ankle dorsiflexion; LKE, left knee extension; RKE, right knee extension.

All TD children were able to generate activation of the primary agonist muscles during each intentional movement task assessed. All TD children were able to produce bilateral muscle activation during bilateral hip and knee movements, neck flexion, and sit up tasks. In unilateral movement tasks (left and right knee extension and ankle dorsiflexion), EMG patterns ranging from bilateral muscle activation to isolated muscle activation around a single joint were observed (Figure 2). Generally, the pattern of EMG activity included activation of multiple muscles distant and/or contralateral to the joint being moved in younger TD children, and more isolated to primary agonists and antagonists in older TD children, with moderate and statistically significant correlations found between age and qualitative categorization for all unilateral movement tasks (left ankle dorsiflexion, correlation coefficient (CC) = 0.472, *p* = 0.023; right ankle dorsiflexion CC = 0.468, *p* = 0.021; left knee extension CC = 0.708, *p* = 0.0002; right knee extension CC = 0.624, *p* = 0.0015). Figure 2B shows the relationship between TD age and qualitative categorization for all unilateral movement tasks.

### Muscle Activation Patterns Below the Lesion in Children With Spinal Cord Injury

**Figure 3:**
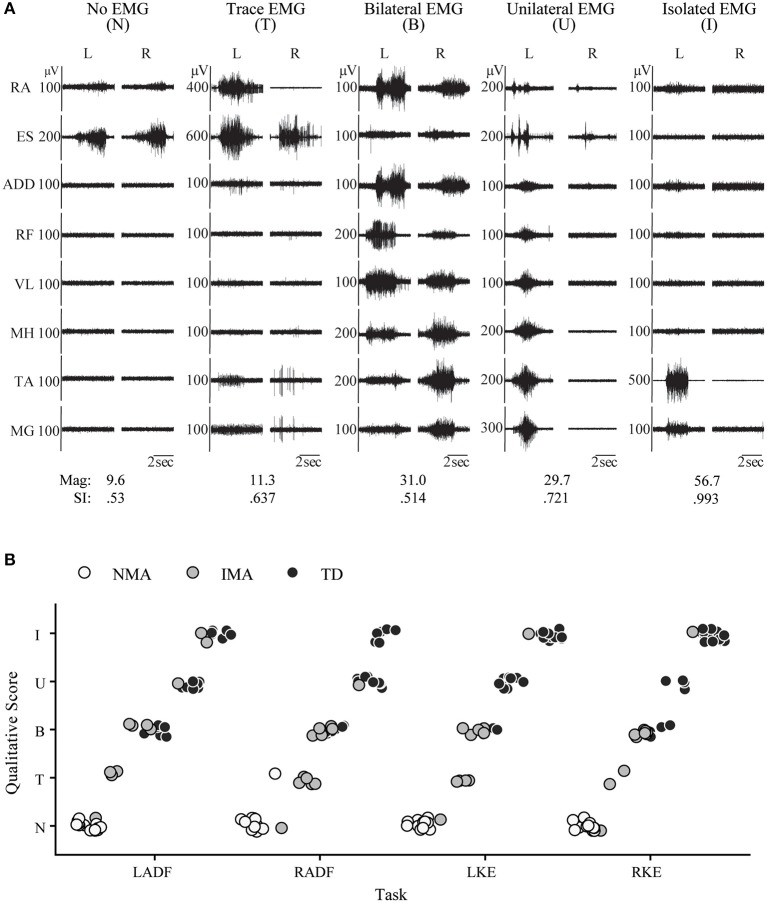
Pediatric functional neurophysiologic assessment: Qualitative and quantitative evaluation of residual motor patterns in children with SCI. **(A)** EMG recordings from 5 different children with SCI during performance of the left ankle dorsiflexion task, demonstrating the 5 muscle activation patterns observed during development of isolated muscle activation in these children: no muscle activation, trace muscle activation, bilateral muscle activation, unilateral muscle activation, and isolated muscle activation. RA, rectus abdominus; ES, erector spinae; ADD, adductor magnus; RF, rectus femoris; VL, vastus lateralis; MH, medial hamstrings (semitendinosus); TA, tibialis anterior; MG, medial gastrocnemius. SI and magnitude values corresponding to each example. **(B)** Relationship between group (NMA, IMA, and TD) and muscle activation pattern for each unilateral movement task, N, no activation; T, trace activation; B, bilateral activation; U, unilateral activation; and I, isolated activation; IMA, intentional muscle activation; NMA, no muscle activation; TD, typically developing.

In addition to the EMG muscle activation patterns observed in TD children, two other patterns were observed in children with SCI (Figure 3A). The first of these was the complete absence of movement-related EMG activity in any leg muscle. In 12/24 SCI children, no EMG activity in leg muscles was observed upon initiation of the movement attempt during any movement task (qualitative score = N). These children were considered as the no intentional muscle activation (NMA) group for subsequent analysis. Twelve children with SCI were able to repeatedly produce EMG activity at the onset of intentional movement attempts in at least one leg muscle and movement task (Table 3). These children were considered as demonstrating intentional leg muscle activation (IMA) and grouped for further analysis. Subjects in the IMA group demonstrated a wide range of motor output during intentional movement trials. Among those demonstrating intentional muscle activation, trace activation occurred in 32% of movement tasks, bilateral activation occurred in 49% of movement tasks, while unilateral or isolated activation occurred in 4% of movement tasks each. In contrast to typically developing children, no correlation with age was observed for any event. There was no relationship found between presence of intentional movement and mechanism of injury (*p* = 0.205). Figure 3B gives the average qualitative description for each event for the NMA, IMA, and TD groups.

**Table 3:**
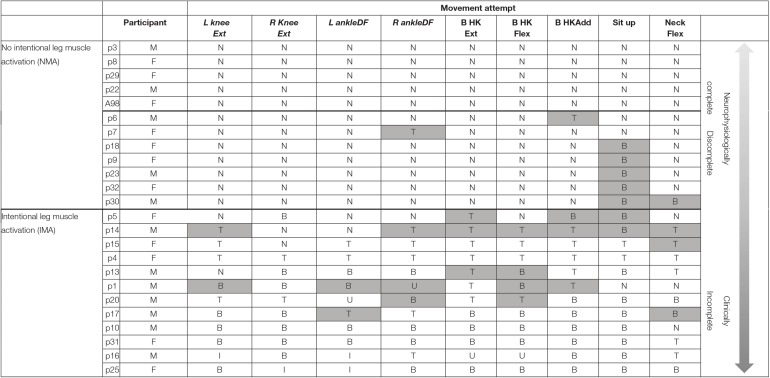
Summary of qualitative muscle activation assessment.

*Qualitative muscle activation scores for all SCI participants for all unilateral (italics) and bilateral movement tasks. N, no activation; T, trace activation; B, bilateral activation; U, unilateral activation; I, isolated activation. Shaded boxes represent delayed-onset (>1 s) muscle activation for a given participant and movement task. The last column gives the average score across all events for each participant; the bottom three columns give the average for each event for the NMA, IMA, and TD groups. NMA, no muscle activation; IMA, intentional muscle activation; TD, typically developing*.

### Quantitative Assessment of Muscle Activation Patterns in TD and SCI Children

**Figure 4:**
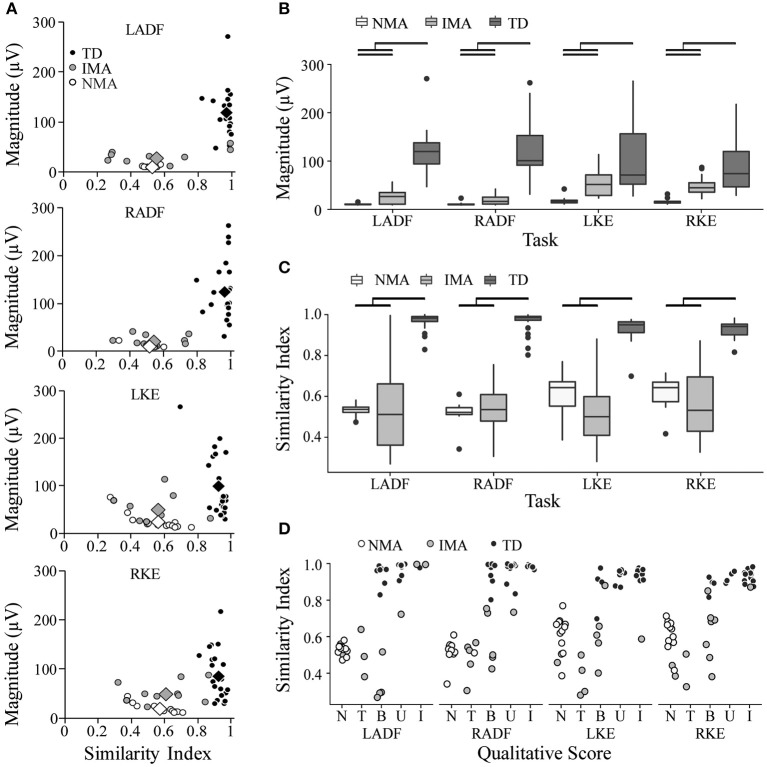
Summary of quantitative evaluation of unilateral movement tasks in TD children and children with SCI. **(A)** SI and Magnitude values are plotted against each other for comparison of group results for each unilateral movement task. Circles represent individual participants; diamonds represent group averages. **(B)** average magnitude values by group **(C)** average SI values by group **(D)** Comparison of SI values and qualitative score. Black bars indicate statistically significant differences between groups (*p* < 0.05). SI, similarity index; LADF, left ankle dorsiflexion; RADF, right ankle dorsiflexion; LKE, left knee extension; RKE, right knee extension; IMA, intentional muscle activation; NMA, no muscle activation; TD, typically developing.

For quantitative assessment of muscle activation patterns, PRVs were generated for each event, using the RVs obtained from TD children (see Methods). The PRVs were then used to compute the VRI for all children (see Magnitude and SI values in Figures 2, 3). Figure 4 summarizes the VRI results for all unilateral movement tasks. SCI participants in both the IMA and NMA groups had significantly lower magnitudes and SI values in comparison with TD children (*p* < 0.01). As expected, magnitude was also significantly different between the NMA and IMA groups for all tasks (*p* < 0.05), with little variability in magnitude or SI values in the NMA group due to the lack of EMG activity in these participants. In TD children, SI values were close to one for each task, and were not significantly correlated with age. In contrast, significantly greater variability in SI values was observed for IMA participants (*p* < 0.0001) (Figure 4B).

Table 4 summarizes the VRI results for all movement tasks. Across all tasks, SI values were significantly larger in the TD group as compared with either the NMA or the IMA groups (*p* < 0.01), however no significant differences were observed between the SCI groups. With regard to magnitude, significant differences were found for all events and all groups, with the exception of NF (IMA vs. TD) (*p* < 0.05), wherein NMA magnitude values were significantly lower than all other groups. IMA magnitudes were significantly larger than the NMA group, and significantly smaller than the TD group. A positive correlation was found between SI and qualitative scores across all tasks (CC = 0.67, *p* < 0.0001) (Figure 4D, and see examples in Figures 2A, 3A).

**Table 4:** Range of SI and magnitude values for the nine tasks performed by the injured and control (typically-developing) groups.

	**Similarity index**						**Magnitude of RV (μV)**					
	**SCI**				**TD**		**SCI**				**TD**	
	**NVM**		**VM**				**NVM**		**VM**			
	**Mean ± SD**		**Mean ± SD**		**Mean ± SD**		**Mean ± SD**		**Mean ± SD**		**Mean ± SD**	
**Task**	**Min**	**Max**	**Min**	**Max**	**Min**	**Max**	**Min**	**Max**	**Min**	**Max**	**Min**	**Max**
LADF	0.43 ± 0.097		0.47 ± 0.28		0.95 ± 0.053		22.19 ± 11.87		57.25 ± 30.46		123.83 ± 48.42	
	0.27	0.54	0.21	0.99	0.78	0.99	12.76	47.78	33.71	129.51	48.02	271.54
RADF	0.41 ± 0.072		0.40 ± 0.19		0.94 ± 0.076		21.20 ± 9.98		54.57 ± 37.23		130.19 ± 62.04	
	0.27	0.50	0.22	0.75	0.69	0.99	12.71	42.21	25.04	137.81	33.30	262.09
LKE	0.57 ± 0.14		0.54 ± 0.17		0.93 ± 0.060		22.85 ± 16.98		53.73 ± 29.37		98.68 ± 65.32	
	0.28	0.77	0.30	0.88	0.70	0.97	11.27	74.78	23.69	113.39	28.03	265.15
RKE	0.58 ± 0.11		0.60 ± 0.19		0.92 ± 0.039		19.40 ± 9.53		52.40 ± 21.75		86.32 ± 49.27	
	0.38	0.71	0.32	0.87	0.81	0.98	11.14	44.03	22.44	87.00	29.35	217.22
BHKF	0.75 ± 0.12		0.72 ± 0.11		0.90 ± 0.063		23.42 ± 13.68		82.55 ± 53.87		171.67 ± 125.70	
	0.53	0.95	0.60	0.91	0.74	0.96	11.37	59.04	30.72	184.10	57.52	602.64
BHKE	0.74 ± 0.11		0.66 ± 0.11		0.92 ± 0.035		28.13 ± 23.18		93.17 ± 62.81		204.06 ± 88.56	
	0.51	0.90	0.53	0.82	0.84	0.99	11.31	92.98	30.93	235.12	96.25	407.91
BHA	0.76 ± 0.14		0.69 ± 0.14		0.89 ± 0.059		24.98 ± 15.50		75.37 ± 46.03		124.48 ± 63.41	
	0.52	0.95	0.51	0.96	0.74	0.97	11.35	60.70	34.03	186.63	39.29	238.10
NF	0.53 ± 0.13		0.69 ± 0.23		0.94 ± 0.073		27.94 ± 17.75		95.26 ± 97.77		140.63 ± 107.45	
	0.31	0.74	0.28	0.98	0.67	0.99	12.51	74.02	18.47	294.49	26.61	441.49
Sit up	0.64 ± 0.13		0.70 ± 0.22		0.91 ± 0.067		33.76 ± 32.0		121.40 ± 111.9		197.18 ± 126.23	
	0.36	0.88	0.33	0.94	0.75	0.98	12.69	128.89	35.33	398.96	60.90	580.36

*SI, similarity index; LADF, RADF, left and right ankle dorsiflexion; LKE, RKE, left and right knee extension; BHKF, bilateral hip and knee flexion; BHKE, bilateral hip and knee extension; BHA, bilateral hip adduction; NF, neck flexion*.

### Delayed Muscle Activation Below the Lesion in Children With Spinal Cord Injury

**Figure 5:**
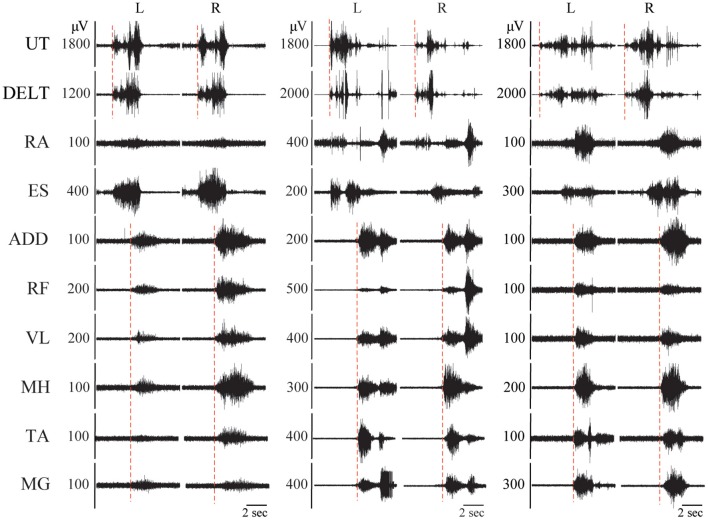
Delayed-onset muscle activation in children with SCI. EMG recordings from 3 different children with SCI during performance of the sit up task, demonstrating delayed onset bilateral muscle activation in all leg muscles. The hashed red lines indicate the timing of onset of muscle activation. UT, upper trapezius; DELT, anterior deltoid; RA, rectus abdominus; ES, erector spinae; ADD, adductor magnus; RF, rectus femoris; VL, vastus lateralis; MH, medial hamstrings (semitendinosus); TA, tibialis anterior; MG, medial gastrocnemius.

In addition, large amplitude, generalized, but temporally delayed activation was recorded in most, if not all muscles below the injury in 19 and 28% of movement trials in the NMA and IMA groups, respectively (Table 3, Figure 5). This EMG activity was not timed to the onset of the movement prompt, but rather always occurred after a delay of at least 1.4 s (average of 5.5 ± 2.9 s) following the child's attempt to move, which can be seen in the figure as the onset of EMG activity in the upper trapezius and deltoid muscles. Delayed muscle activity was not seen in children with the 5 lowest, as well as the 4 highest average qualitative scores (Table 3).

### Trunk Muscle Activation Above and Below the Lesion in Children With Spinal Cord Injury

**Figure 6:**
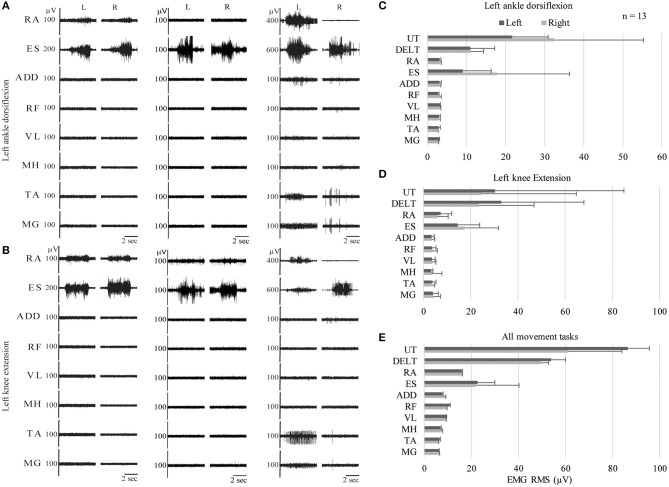
Trunk muscle activation during intentional movement attempts in children with cervical SCI. EMG recordings from three different children with SCI during two different leg movement attempts: left ankle dorsiflexion **(A)** and left knee extension **(B)**. **(C–E)** Average RMS values for left ankle dorsiflexion, left knee extension, and grand average across all movement tasks for all children with cervical SCI. EMG, electromyography; RMS, root mean square; UT, Upper Trapezius; DELT, Deltoid; RA, Rectus Abdominus; ES, Erector Spinae; ADD, Adductor Magnus; RF, Rectus Femoris; VL, Vastus Lateralis; MH, Medial Hamstrings; TA, Tibialis Anterior; MG, Medial Gastrocnemius.

Unexpectedly, we observed activation of the ES muscles, recorded at the T10–T12 vertebral level, in all children with SCI, regardless of injury level, chronicity, or ability to perform intentional leg movements. Activation of the RA muscles was observed in all children in the IMA group, but only low amplitude EMG activity in these muscles was observed in the NMA group- this occurred in 9 instances in 5 participants across all movement tasks. RA EMG activity was frequently observed during delayed onset muscle activation, while ES EMG activity could be observed at the onset of each movement attempt (Figure 6). In Figures 6A,B, examples of EMG activity recorded in three children with cervical SCI, two from the NMA group, and a third from the IMA group, who was only able to produce trace EMG activity. Two different movement tasks are shown, left ankle dorsiflexion and left knee extension. In all 3 subjects, EMG activity can be clearly seen in the erector spinae muscles during the child's attempt to perform the movement. Erector spinae EMG activity can also be seen in the examples used in Figures 3, 4. This contrasted with TD children, who demonstrated little to no EMG activity in the erector spinae or rectus abdominus muscles during the same movement attempts (cf. Figure 2). Cervical SCI group averages of EMG RMS during these two events (Figures 6C,D), as well as averaged across all events (Figure 6E) demonstrate activation of ES, and to a lesser extent RA during movement attempts, despite the distal location and innervation of these muscles relative to the level of SCI.

## Discussion

In the current study we observed a broad range of motor ability across 24 pediatric SCI participants. As has been noted in similar studies of adults with SCI (27, 31, 40), attempts to produce isolated movements frequently resulted in more widespread or generalized muscle activity. In addition to the intentional muscle activity observed, delayed muscle activation was also present, potentially indicating the existence of descending supraspinal input onto the spinal motor circuitry even in children considered as motor and sensory complete on clinical examination (28, 41, 42). Perhaps most interesting, activity in the trunk muscles was observed in all participants, regardless of level or severity of injury. These findings have important implications for rehabilitation of children with SCI.

### Age-Related Differences in Muscle Activation Patterns During Intentional Movements in TD Children

Analysis of muscle activation patterns produced by TD children in the performance of intentional movements of individual limbs or joints revealed age-related differences; specifically, younger children were more likely to produce bilateral muscle activation patterns, while older children were more likely to demonstrate unilateral isolated muscle activity occurring only in the primary agonists and antagonists at a single joint (Figure 2). Further, these differences were most frequently observed during the ankle dorsiflexion task, which requires isolated movement of a distal joint. This movement requires the most selective muscle activation pattern among movement tasks included in the pediatric FNPA, and would therefore be expected to be the most difficult to perform from a developmental perspective. As was expected, there was a greater range of SI values for TD children when compared with non-injured adults reported in these earlier studies. This is likely due to the relatively large interindividual variability in motor patterns present in young children (12, 16, 20, 43).

Among non-injured populations, the generation of bilateral muscle activity during unilateral movement attempts has been observed both in adults during movements requiring maximal effort to perform or sustain (44), as well as in young children who are still developing movement control strategies (45). A large body of literature discusses the existence of what has most commonly been referred to as associated movements (AMs), referring to muscle activity not contributing to the movement task, which can be described by its duration, intensity, and location (i.e., ipsi- vs. contralateral muscles) (13, 16, 43, 46, 47). Such movements may also be referred to as “imitative synkinesis” (48) or “motor overflow” (17). Bilateral motor activation patterns also are known to occur in adults, but only during fatigue, or when performing a task requiring maximal effort (49). Bilateral muscle activation for a unilateral task as described in the current study appears synonymous with contralateral AMs, which is the most frequently assessed parameter of movement quality in children with motor disorders. The presence and frequency of AMs for a given movement decreases and with increasing age in children, with AMs persisting the longest for the most complex motor tasks. The presence and degree of AMs also varies significantly between children of a given age for a given task, with interindividual variability decreasing with increasing age. This phenomenon is thought to typically resolve in the first decade of life, except in clinical populations (50). A functional MRI study of children with attention deficit hyperactivity disorder who exhibit AMs found that the presence of AMs was correlated with decreased activation of the primary motor and premotor cortical areas during motor task performance (51). The authors hypothesized that the decreased cortical activity resulted in reduced inhibition of unwanted activity during the task. In the case of SCI, the disruption or loss of CST input onto spinal motor pools below the level of lesion would be consistent with this hypothesis.

Similarly, refinement of locomotor muscle activation patterns for walking, beginning with stepping like movements in infants through adulthood has been described (14, 15). Infant muscle activation patterns were characterized by mass unilateral muscle activation corresponding to the stance phase of gait, with co-activation of agonists and antagonists at the hip, knee, and ankle, showing little variation from the start to the end of the step cycle. Analysis of muscle activation patterns among infants, toddlers, preschoolers, and adults, found that locomotor refinement primarily occurs between infancy and pre-school age, with comparatively little change happening between preschool age and adulthood.

The development of refined motor control in the maturing nervous system is associated with the maturation of the corticospinal tract and the arrival of projecting corticospinal tract (CST) axons to their targets within the spinal cord (52). In contrast, postural and locomotor motor behaviors appear earlier in development and may include more generalized and bilateral muscle activity (18, 52, 53). These motor functions are mediated primarily by spinal tracts originating in the brain stem, including reticulospinal and vestibulospinal tracts. In the present context, bilateral or more generalized muscle activity seen in young TD children may reflect the presence of these earlier developing motor control strategies and/or the existence of immature CST-mediated control strategies.

These results suggest that assessment of motor control in children with neurological dysfunction should account for age-related differences. As pediatric motor outcome measures acknowledge the influence of development in skills such as balancing and jumping, neurophysiologic assessments should also acknowledge the influence of development through the use of age-specific standards. Future studies should examine the muscle activation patterns associated with more complex motor tasks, as well as motor tasks not primarily mediated by the CST, such as dynamic posture and gait.

### Patterns of Muscle Activation Below the Lesion in Children With SCI

Both qualitative and quantitative methods of analysis demonstrated clear differences in muscle activation patterns for a given task between TD children and those with SCI. Importantly, EMG assessment allowed objective determination of the presence or absence or intentional muscle activation in children too young to be reliably assessed via the ISNCSCI examination (10). It is notable that patterns of muscle activation in children with SCI appeared similar to that of young children who have not yet developed isolated movement control. The finding of significantly lower SI and magnitude values in children with SCI mirrors those reported in earlier studies of adult SCI (33, 34) and provide objective confirmation of qualitative differences reported.

Children in the IMA group were able to produce EMG activity in muscles innervated by spinal segments below the level of lesion in nearly every trial, demonstrating more consistent motor output (cf. Tables 2, 3). While this motor output was frequently not isolated to the muscles and movements being assessed in a given task, and may be considered as impaired motor function, it is also a clear indication of supraspinal influence on spinal motor output below the level of SCI (41, 42, 44, 54). In the absence of isolated movements, movement or muscle activity which is not isolated to a particular joint should not be disregarded as a “spasm” if the pattern is consistent and repeatable during multiple movement attempts. These features make it apparent that this activity is not reflex driven, but results from the intentional attempt to move, and thus reflects residual brain influence on the spinal motor circuitry.

#### Subclinical Muscle Activation Below the Lesion in Children With SCI

We observed muscle activation patterns reminiscent of those produced during so-called “reinforcement maneuvers” described in similar studies of residual motor function in adult SCI. These patterns have been postulated to result from subclinical residual brain influence on spinal motor circuitry (28, 42, 54). The features of the delayed-onset muscle activity responses were strikingly similar in terms of time-delay from the onset of the movement attempt, as well as the amplitude and duration of activity. As has been reported in studies of adult SCI, these responses were seen in children with SCI with and without the ability to perform intentional movements, but were not seen in typically developing children (cf. Table 3).

#### Muscle Activation in the Erector Spinae Muscles Below the Lesion After SCI

The finding that the erector spinae muscles remain under descending influence after otherwise motor complete SCI is surprising; but has been reported after adult SCI as well (55, 56). Little is known about the effect of SCI on the axial musculature innervated by the thoracic segments, as the ISNCSCI exam only assesses key muscles in the extremities. In the current study, the erector spinae muscles, recorded at the T10 vertebral level, became active at the onset of any movement attempt in children with SCI, as was observed for muscles innervated by spinal segments above the level of SCI, regardless of whether or not muscle activity was generated in leg muscles (Figures 3, 5, 6). The T10 vertebral level corresponds with the T12 spinal segmental level. This was below the level of spinal lesion in 21/24 patients (Table 2). In contrast, trunk muscle activation was only seen in TD children during proximal limb movements which would require proximal stabilization of spine and not seen in the distal ankle dorsiflexion task. The ankle dorsiflexion task was performed with the limb fully supported by the bed, meaning effective performance of the movement did not require stabilization of the spine. The consistent activation of erector spinae muscles during all movement tasks in children with SCI may be a result of the increased effort necessary to perform movements which require activation of muscles innervated below the level of injury, in which these children had compromised function.

As it relates to measurement of residual motor function, the current approach may effectively complement the ISNCSCI exam, filling a gap in currently available measurements. The ISNCSCI exam targets isolated limb movements and muscle activation thought to be mediated by the corticospinal tracts (57), and excludes testing of axial muscles innervated by the thoracic segments, such as the rectus abdominus and thoracic erector spinae muscles. In contrast, the FNPA includes assessment of the trunk musculature during both direct and indirect (i.e., trunk vs. limb) movement tasks.

### Significance of Residual Motor Function Below the Lesion After Pediatric SCI

In children with SCI, presence of more generalized muscle activation, ES muscle activation, and the relative absence of more refined, isolated motor patterns may reflect a preferential sparing of descending pathways with more spatially diffuse and numerous projections within the thoracolumbar spinal cord, such as the reticulospinal tracts (58, 59). The reticulospinal tracts are involved in controlling posture and locomotion, and contribute the majority of descending influence on the axial and proximal limb motor pools via bilateral projections (60–62). Although these descending projections originate in the brain stem, the nuclei from which they emerge receive inputs from the sensorimotor cortex, thus providing a potential indirect route for intentional movement commands from the brain to descend to the spinal cord. It is noteworthy that the majority of descending fibers found below the cervical enlargement are either reticulospinal or propriospinal (58, 63). In the event the corticospinal tracts are disrupted, a variety of animal models have demonstrated that these pathways, as well as others (i.e., propriospinal tracts) can mediate functionally meaningful restoration of cortical control of motor function below the lesion (64–68).

Alternatively, the erector spinae muscle spans the length of the spinal column, and is innervated by nerve roots from the adjacent spinal segment (69). Following SCI, one could expect the erector spinae to remain innervated immediately above the level of SCI. The presence of innervation and active muscle contraction above the level of injury might conceivably propagate through the muscle, inducing contractions below the level of SCI. Future studies should further investigate this phenomenon; recording from the erector spinae at multiple spinal levels would allow observation of the timing of muscle activity in erector spinae both above and below the level of lesion.

### Implications for Pediatric SCI Rehabilitation: Identifying Opportunities for Recovery

The generalized muscle activation patterns, as well as the apparent preservation of postural extensor muscle activation which was observed after pediatric-onset SCI in the current study reflects residual supraspinal influence on spinal motor circuits, with important implications for pediatric-onset SCI rehabilitation. A recent case study describing the recovery of intentional leg movement in an individual with a chronic, motor complete SCI reported that patterns of muscle activation which transitioned from no intentional muscle activity, to generalized and bilateral muscle activation, and finally to an isolated muscle activation (23). Similarly, an earlier study of motor recovery after acute SCI also described a similar progression in muscle activation patterns across the recovery period (27). Therefore, regardless of the descending systems involved, the presence of these impaired motor patterns may provide an indication of the potential for future motor recovery. Several recent studies of intensive, activity-based locomotor training in combination with electrical spinal stimulation demonstrate the ability to promote functionally relevant neuroplastic changes in the quality of both intentional and postural spinal motor output, after chronic, motor complete SCI (24, 26, 70–73).

Of specific importance for rehabilitation clinicians, the results of the current study suggest that in children, significant potential may exist for recovery of motor function, particularly in the postural extensors. The erector spinae muscles are primary agonists for spinal extension, in addition to being responsible for maintenance of upright posture via spinal stabilization during standing and walking, as well as during intentional limb movements. Thus, these muscles may be activated by both intentional and automatic processes. Conventional pediatric-onset SCI rehabilitation largely assumes no potential for upright sitting for children with cervical or upper thoracic lesions (74, 75). A recent study of 217 children with SCI found that 100% developed scoliosis with a minimum cobb angle of 10 degrees (76). Perhaps more importantly, no correlation was found between scoliosis and injury level or severity, with only age at injury being predictive of scoliosis severity, i.e., need for spinal fusion. Although it is assumed that paralysis of the trunk muscles is the cause of scoliosis after pediatric SCI, no prospective studies have been undertaken to provide causal evidence.

A recent study of children with pediatric-onset SCI, investigating the responsiveness of an outcome measure designed to measure trunk control in children, reported improved trunk control in all participants, regardless of chronicity, initial impairment, or prior therapeutic intervention, following 3 months of participation in activities-based locomotor training (21). Whether the ability to produce muscle activity in the thoracic extensors after SCI demonstrated in the current study is related to these improvements is a critical question which deserves further study. Not only does the potential for improved trunk control hold obvious importance for general mobility and independence, but considering the startling rate of neuromuscular scoliosis in pediatric SCI, the potential to mitigate this risk may prove equally important as the child ages. Any opportunity to reduce or prevent the progression of neuromuscular scoliosis would result in a reduction in additional hospitalizations, surgeries, and the associated physical, emotional, and financial challenges for children and their caregivers (76–79). Therapists and researchers may consider the use of the FNPA to document ES activation after pediatric SCI. Knowledge of such activation can justify therapeutic intervention aimed at improving intrinsic postural control as opposed to teaching compensatory balance strategies during functional sitting tasks.

### Limitations

A potential limitation to this study is that we utilized a convenience sample, resulting in heterogeneity among SCI etiologies included. However, no relationship between presence or qualitative nature of intentional muscle activation and injury etiology was observed. This diverse sample may not have readily influenced the outcome of the study. Our sample included children with both traumatic and non-traumatic injuries and of varying etiology even within these categories. Other aspects that may have influenced whether EMG activity was observed below the lesion may be further considered. Neuroimaging techniques could also be utilized to further clarify the location and severity of the spinal lesions in the study participants. Given the surprising findings in the current study, MRI–based localization of lesions within the spinal cord- in particular, cross-sectional images at the level of lesion- may provide additional data for analysis. Such information could more accurately define the study population and add additional insight to the potential anatomical explanations for the results.

## Conclusions

In children, residual muscle activation in trunk and leg muscles below the level of SCI observed during intentional movements and attempts, regardless of the level of coordination or selectivity in the resulting motor output, may be considered evidence of residual descending influence on spinal motor circuits after injury. Further, the presence of such residual muscle activation, regardless of its immediate functional relevance, may provide an objective indicator of the potential for further recovery of postural and/or intentional motor function. Postural and intentional movement attempts, performed repeatedly, and in the presence of appropriate proprioceptive cues, should be a focus of therapeutic intervention for children with any amount of residual motor function. Evaluation of multi-muscle activation patterns during intentional movement attempts in children with SCI was found to be valuable in the pediatric population, as it allowed characterization of residual motor function based on objective EMG recordings, was not dependent upon the observational skills of the assessor at the time the movements were attempted, and could be conducted in children as young as 3 years old.

## Future Directions

Further study is required to determine whether and to what extent nervous system development at the time of injury influences recovery after pediatric SCI; a child injured prenatally or in the first year of life has not yet developed the control strategies required for the execution of movement patterns produced by older children and adults. In addition, we were unable to complete the pediatric FNPA protocol in children younger than three, and determination of residual motor function after SCI in this population remains challenging.

## Data Availability Statement

The datasets generated for this study are available on request to the corresponding author.

## Ethics Statement

The studies involving human participants were reviewed and approved by Institutional Review Board, University of Louisville, IRB approval #15.0183. Written informed consent to participate in this study was provided by the participants' legal guardian/next of kin.

## Author Contributions

DA and AB contributed to the study design. DA, LM, NG, and AB participated in data collection, analysis/interpretation, and manuscript preparation. SA and BU participated in data analysis/interpretation and manuscript preparation.

### Conflict of Interest

The authors declare that the research was conducted in the absence of any commercial or financial relationships that could be construed as a potential conflict of interest.
